# Philadelphia Hospital

**Published:** 1838-01-31

**Authors:** 


### PHILADELPHIA HOSPITAL.

*Saturday, 6th January.*—Dr. Gibson commenced the lecture by exhibiting two patients; one with sloughing ulcer of the leg near the knee, of great extent, the other with chronic gangrene of the foot. After describing the appearances, and pointing out the peculiarities of the sloughing ulcer, Dr. Gibson said:

The very best treatment that you can prescribe internally, is ammonia. The stimulation produced by this article is of a different character from that which is brought about by the use of brandy, whiskey, and other alcoholic stimuli, and which are followed by a corresponding state of depression. I have known patients become so weak from the exhibition of these articles, as to sink under the effects of them. Not so with ammonia; although a powerful excitant, its influence is of a more permanent nature. It is particularly adapted to states of depression, occasioned by violent shocks to the nervous system, by the immoderate use of evacuants, by the wearing out of the system from disease, or by severe operations. On these occasions, I have seen more advantage derived from the administration of ammonia, than from that of any other set of remedies. Sir Astley Cooper is of opinion, that it is the very best medicine that can be given internally for such cases. This is the result of my own experience, and I was informed by Dr. Physick, that he had the same confidence in its powers, and was in the habit of resorting to it, under the circumstances which I have described. If any thing can save this man, and make an impression upon the slough, it will be the vigorous use of the ammonia, combined with the sulphate of quinine, or camphor and opium. As a general rule, local applications are of little service in cases of this kind; your main reliance must be upon internal remedies. Yeast, and fermenting poultices are useful, in keeping the parts soft, and free from fœtor: but, they answer no other good purpose. To destroy the offensive smell, attendant on sloughs like this, the use of powdered charcoal has been recommended. The objection to it is, that it reduces the appearance of the surrounding parts to that presented by the slough itself, and makes it difficult to mark the progress of the disorder.

If you meet, then, with such a sore, as that which is now presented to you, rely principally on internal remedies such as I have mentioned. To the use of the fermenting liquors, as beer, porter, brown stout, &c., I have as strong objections, as those which I have expressed with regard to ardent spirits. Sir Astley Cooper says, that these fermenting liquors are more injurious to the constitution, than any single article with which he is acquainted. If you bear in mind the various drugs, such as horse-aloes, and a long list of others, which I might enumerate, which brewers (in Europe at least,) mix with their commodity, you cannot be surprised at this. Even where it is the best and purest in its quality, Sir Astley considers it pernicious. He seldom performed an operation on a confirmed beer-drinker, a brewer's servant or apprentice for example, (who of course swallow large quantities of these liquids) that proved successful. Wine, and even brandy, and articles of that description, I should resort to in preference to the class of liquors against which I have just been cautioning you. I dwell upon this subject, because there exists a very general impression that malt liquors are especially adapted to cases of debility. This is a mistake: they fill the patient up with air, and make him soft and frothy, but give him no real strength.

The brilliancy of this man's eye might lead you to suppose that he was by no means in the dangerous state in which I have depicted him. This however is the eye which you meet in with in mania-a-potu, and is very far from being an indication of health. It may co-exist with a very alarming degree of prostration. I have seen it in this state, ten or twenty minutes before dissolution. There is a bare possibility that this man may recover, but it is highly improbable. After the long period that the disease has continued, and at his age, it will be no easy matter to get him on his legs again.

The patient, whom I next present to you, offers a specimen of a disease, usually peculiar to old age, although I have met with it in individuals, from twenty to twenty-five years of age. It is called by Pott, mortification of the toes and feet; I shall apply to it the term, chronic mortification. It generally attacks individuals, who have reached their sixtieth year, making its first inroads upon one or more of the toes. A small blue spot appears upon one of these, which gradually creeps up the lower extremities. Sometimes, one or two little marbled spots are seen on a toe, which gradually run into each other, then make their way to the ankle, and finally involve the leg. Usually, before the disease reaches the middle of the leg, or even the ankle, the patient is worn out, from its effects. At other times, where the constitution is good and the patient not too old, the toe drops off, the sore heals up, and recovery follows.

This individual offers a very fair example of the disease. The line, separating the healthy from the diseased parts, is well marked; there are a few healthy granulations thrown out.

It has been said that chronic mortification is invariably connected with an ossified, or otherwise diseased state of the arterial system. By some, it has been attributed to a deposit of calcareous matter in these vessels. In most individuals, who have attained the age of fifty, the internal, and particularly the middle coats of the arteries, are apt to be involved in this calcareous change. Those in the neighbourhood of the wrist, and having their origin, very near the heart, are principally affected. The subclavian artery I have known to be filled with bony matter. The arteries of the arm, usually escape: but those of the forearm, the radial, interosseous, and ulnar, are often attacked. Whether the disease under notice be invariably the result of this morbid state of the arterial system, I am not prepared to decide. I rather conclude, there has been some mistake on this subject, in attempting to exclude all other sources of the disease.

This man is 72 years of age. His pulse can scarcely be felt at the wrist, and is extremely wirelike.

The treatment has been very similar to that pursued in the last case. The system has been supported by bark, wine, ammonia and even brandy. In addition, a fermenting cataplasm has been applied for the last few days, to keep the part moist and clean. There is however no probability, scarcely a possibility of his recovery. It is a disease which is very seldom cured, even in the young, and this man's very advanced age seals his fate. In young persons, I have seen the application of a blister occasionally of service; but generally it is of no service. A slough may be produced, and thrown off; healthy granulations appear, and the parts cicatrize. But even when this has taken place, the disorder is very apt to return.

Dr. Gibson next took up the subject of fracture of the clavicle, and described and applied Dessault's apparatus for the treatment of this accident. This apparatus is so entirely familiar to the surgical world, that we forbear entering at length into Dr. Gibson's remarks. He took occasion to express his undiminished confidence in the plan of Dessault, as originally suggested by that distinguished surgeon, from its simplicity, the ease with which the materials for using it may be procured, and the general success which attends its application.

He contended, however, that it was perfectly immaterial whether the surgeon used the particular bandages of Dessault or any other form of apparatus, provided the *principles* of that surgeon, (principles universally acknowledged to be mathematically correct,) were kept in view. Lastly Dr. Gibson called upon the students themselves to apply the bandage, which operation was performed fifteen or twenty times before the class, and in general, with great accuracy and precision.

---

NEURALGIA.

Dr. Jackson followed Dr. Gibson.—I shall to-day, gentlemen, call your attention to, and illustrate the diseases of the nervous system. They are very numerous and very obscure; difficult to distinguish; to diagnosticate; often confounded with other affections; difficult of management; doubtful as to the proper method of treatment, harrassing to the patient, and frequently defying all the best directed resources of medical art. That class of nervous diseases, which I shall at present more particularly bring under your notice, are the neuralgia. This term is derived, as you know, from two Greek words—νευρος and αλγος—and signifies *nerve—pain.* We shall bring forward several cases, now in the house, to illustrate some of the innumerable varieties of this affection. Previous to this being done I will give you a general and rapid sketch of the nervous system. It is important that you should fully understand the functions and structure of this portion of the animal economy, before you take up pathological deviations that belong to it. I shall then proceed to the symptoms, causes and treatment of neuralgia. At the present day pathology rests upon the organic structure. Wherever our knowledge of the anatomy and physiology of a part is most perfect, we better understand the abnormal modifications to which it is subject. Let us build our pathological views on this foundation and they will be more accurate. Our investigation into the structure, functions, and diseases of the nervous system are making rapid strides, and I have but little doubt that, in all probability, it will be the first part of medicine to be completed.

The nervous system is entirely dissimilar to every other portion of the animal economy. It differs in its organic elements, which is peculiar to it; in its organic structure; in its mode of vitality; and in the character of its functions. It is so far separated from the other portions of the organization, especially the cerebro-spinal system—enclosed in the cranium and spinal column, which may be regarded as its skeleton—that it may, without a forced analogy, be looked upon as a distinct animal—the *nerve animal*—engrafted on the trunk or vegetative animal, overruling and directing its functions and actions.

The nervous system presents two great divisions. 1st. The ganglionic, connected with the trunk or vegetative animal, exciting, regulating, co-ordinating the functions of organic life—nutrition and the secretions: 2nd. The cerebro spinal, or proper nerve animal, connected with sensibility and the senses; with consciousness, volition and voluntary movement; and with the intellectual and moral faculties.

It is this last which requires our attention at present with respect to its arrangement and functions.

The cerebro-spinal nervous apparatus consists of various portions. They are as follows. *a.* Peripheral or nervous expansions, spread on the surfaces, or interwoven in their tissue, as the skin, mucous membrane, and the organs of the external senses. *b.* The nerves communicating with the spinal cord, or the medulla oblongata. *c.* Spinal cord and medulla oblongata. *d.* The brain, and cerebellum. Now these different portions are formed of two distinct kinds of nervous substance—the grey or pulpy, and the medullary or fibrous. But we cannot detect in the different portions of the nervous system any palpable difference between these substances. Every where they present nearly the same characters. The arrangement or disposition alone is different. Yet we find the most diversified functions, powers wholly dissimilar possessed and exercised by the different portions of this nervous arrangement. How it is that powers so distinct are generated, and exerted by a matter with but little difference in its nature or characters, is wholly inexplicable. It is one of the most profound mysteries of nature, with the solution of which we have nothing to do, our business is confined to the facts developed by observation.

In the higher animal organizations, and the perfect development of the nervous system, anatomical investigations throw but little light upon the intimate arrangement of the nervous structure, its independent centres, or organs, and the mode of their combination. Physiological and pathological phenomena, however, prove that numerous centres of action do exist, and that they are productive of actions and phenomena peculiar to each. Although the demonstration cannot satisfactorily be made in the higher orders, it is accomplished in the lower orders of animals. Now in the higher orders there is always a repetition of what exists in the lower, with some modification of arrangement, and it is thus that we can establish the fact of independent centres in the higher orders. Before proceeding let us be understood in using the term nervous centres. By this term is meant to be designated nervous masses—ganglions—with which nerves communicate, to which impressions are transmitted from the surfaces, external and internal, and which again reflect them, or transmit new and independent excitation into the organs with which they are in connection. Wherever this combination can be shown, there exists a nervous organ, centre, or ganglion.

If you examine in a cursory manner a simple nervous cord, you would suppose it single, on the contrary it is composed of numerous filaments, and it possesses entirely different properties, as we know from its functions being excitative of sensation and muscular contraction. The same is true of the spinal cord itself. This has been fully demonstrated by Sir Charles Bell, Magendie, and a host who have followed them. But there are other divisions than that into posterior cords for sensation, and anterior for motion. This second subdivision is into centres, or the segmentation of the spinal marrow, each segment constituting a centre, and corresponding to the different vertebræ. This we are unable to demonstrate, as has been observed in the higher order of animals; lower in the organic scale we have abundant proof of its existence.

But besides the division of the spinal marrow and brain into centres and segments, there is another division into two lateral halves, this is also demonstrated in the inferior order of animals, and in fœtal development. We have thus a compound action going on in the formation of the nervous system. First, one from the centre to the circumference; second, from below upwards, forming the segments in succession. In the lower orders of animals, and in the fœtus, there exists an elongated vesicle or canal for the spinal cord; and three vesicles at the superior extremity; the first for the bulb of the medulla oblongata; the second for the optic lobes, and the third for the cerebral hemispheres. The nervous substance is first formed on the sides of these vesicles, and increases towards the centre, until the two halves meet and coalesce. The spinal cord closes first at the spinal end, and is formed from below upwards; the hemispheres are the last to be completed. We have thus two distinct cerebro-spinal nervous systems; a right half and a left half, dividing the individual in two. In the human fœtus of five weeks, the spinal cord and brain exhibit the same formation as in the tadpole of fifteen days, and the sheep of the same number of weeks. In the spinal marrow we have said, that there exists numerous centres or ganglia. These ganglia in the mammalia have coalesced in their development, and they form an apparently solid and continuous mass. Now the demonstration of this, it has been asserted, is to be found in the lower animals. The leech exhibits to a remarkable degree this arrangement. This animal consists of numerous segments, each one of which has its separate ganglion, from which proceed nerves that supply the segment; a separate circulatory apparatus, and organs of nutrition. Each segment is then to a certain extent, a distinct animal. The same segmentation is manifested in insects, and in them as they pass through different phases of organization, in their metamorphosis from the caterpillar to the chrysalis and perfect insect, we observe the ganglia of the spinal cord separated from each other in the caterpillar, and then coalesced united two, three and four into single masses, forming centres for the brain and senses, and for the nerves passing to the limbs, thorax and abdomen.

In the higher animals, without detaining you for farther evidence of the fact, the spinal cord is composed of numerous centres, through each of which is transmitted through the nerves that communicate with it, specific powers or excitations. Through some nervous filaments, motility exciting muscular contraction; through others, sensibility by means of which sensation is exerted; others nutritive excitation, determining the nutrition of the part, its circulation and temperature. This last is doubtful as a separate, independent power, but is probable from facts it would be out of place to indicate here, and may be exercised in connection with the centres of the ganglionic nervous system.

Though numerous distinct centres thus exist in the spinal cord, yet they are all united the one with the other, and each with the brain, so as to produce unity of action. This connection of the various centres with the brain is finely illustrated in a case detailed by Magendie. A person in Paris whose case had considerable notoriety, was paralysed in the superior extremities, but had perfect control over the lower ones. He was very active, and it is said much devoted to the gentler sex. This state continued some years; I believe seven. On his death it was ascertained that the central portion of the spinal cord, where the trachial nerves are given off, was disorganised, and in a softened condition; while the exterior strands of fibrous or medullary substance retained its natural structure. Thus the nervous centres which distributed power to the arms being disorganised, they were in consequence paralysed. The centres below were unaffected, and as the connection between them and the cerebral centres was maintained by the fibrous or medullary strands remaining in a state of integrity, volition would call these energies into action.

It was once supposed that in order to cause muscular contraction by external irritation that the compression should be first transmitted to the brain. This however it has been ascertained is not correct. Dr. Marshall Hall, of London, has treated of this subject very fully, and has advanced a number of very interesting views on the reflex action of the nervous centres. I refer you to his work for a demonstration of this reflex action. Dr. Graves of Dublin, Müller of Germany, and Mayo of London, have also put in their claim to this discovery. I might assert mine also, for seven years ago I taught and published the same principle. The view I took of the reflex action was limited to the medulla oblongata, but the principle is the same. I endeavored to establish its existence from the fact of tickling exciting convulsions, and from the production of fatal convulsions being excited by the presence of worms in the alimentary canal, and of indigestable food in the stomach, as hot bread, fried eggs, &c.; articles which in themselves are not endowed with any active properties exerting a specific or injurious influence on the system. The same discoveries are often promulgated about the same time by different persons, occupied in the same order of research, where one could not have had any knowledge of the proceedings of the other. It is certain that in the present instance, Marshall Hall has more fully developed these views than any one else. Müller has extended this doctrine. He asserts that muscular contraction reacts on the sensibility, and that a spasm of a muscle, or its contraction, may originate pain. I have met with one instance which appears to sanction this view. The patient had neuralgia of the spermatic cord, but he suffered only when he was in an erect position. The moment he assumed the recumbent posture he had ease. The contraction of the suspensory muscle of the cord, or of the abdominal muscles seemed here to excite pain, whilst their relaxation gave immediate relief. This solitary instance which I have met with, bears I think on the view taken by Müller.

[We are obliged to postpone from unavoidable circumstances, the remainder of this interesting lecture until our next.]

